# Editorial: Methods and applications in cognitive science

**DOI:** 10.3389/fpsyg.2023.1321214

**Published:** 2023-11-22

**Authors:** Prakash Padakannaya, Elisa Puvia, Radwa Khalil

**Affiliations:** ^1^Department of Psychology, CHRIST University, Bengaluru, India; ^2^Department of Psychological and Social Sciences, John Cabot University, Rome, Italy; ^3^School of Business, Social and Decision Sciences, Constructor University, Bremen, Germany

**Keywords:** methods in cognitive science, event-related potential (ERP), drift-diffusion model (DDM), evolutionary game theory, embodiment and virtual reality (VR), mobile brain and body imaging (MoBi), motor imagery training, inhibitory control

Cognitive Science presents an evolving multidisciplinary discipline aiming at exploring, examining, and explaining cognition in its fullest nuances – its representation, expression, and interplay with internal and external aspects of behavior. The ultimate goal is to achieve a comprehensive, universal, and simultaneously context-specific, probably pluralistic understanding of behavior in its broadest sense. In other words, Cognitive Science aims at unraveling mysteries of intricate interactions between brain-mind-culture/environment and building and blending pluralistic theories following multiapproach and multi-methods. Though this sounds more utopic, the rapid developments in cognitive science testify that we are marching in the right direction and the research findings in cognitive science reveal new facets of (near)truth hitherto unknown. This is happening through the cross-breeding of research methods and approaches from behavioral sciences (psychology, economics, etc.) through life sciences (neuroscience) and physical sciences (quantum mechanics, neurochemistry, etc.). We planned for this special volume on the topic, “*Methods and applications in cognitive science*” to highlight some of such novel methodologies or paradigms that may potentially inspire future research and interdisciplinary collaborations. We invited all types of articles, reviews, and novel protocols that have relevance to the basic philosophy of cognitive science. This special topic series comprises eight full papers that went through the normal peer-review process successfully. We give an overview of these eight papers here following which we reflect on the overall limitations and merits of this Research Topic, and how they could potentially broaden the breadth and depth of inquiry in cognitive science research. Figure 1 presents a schematic representation of the topics covered in this special series.

**Figure 1:**
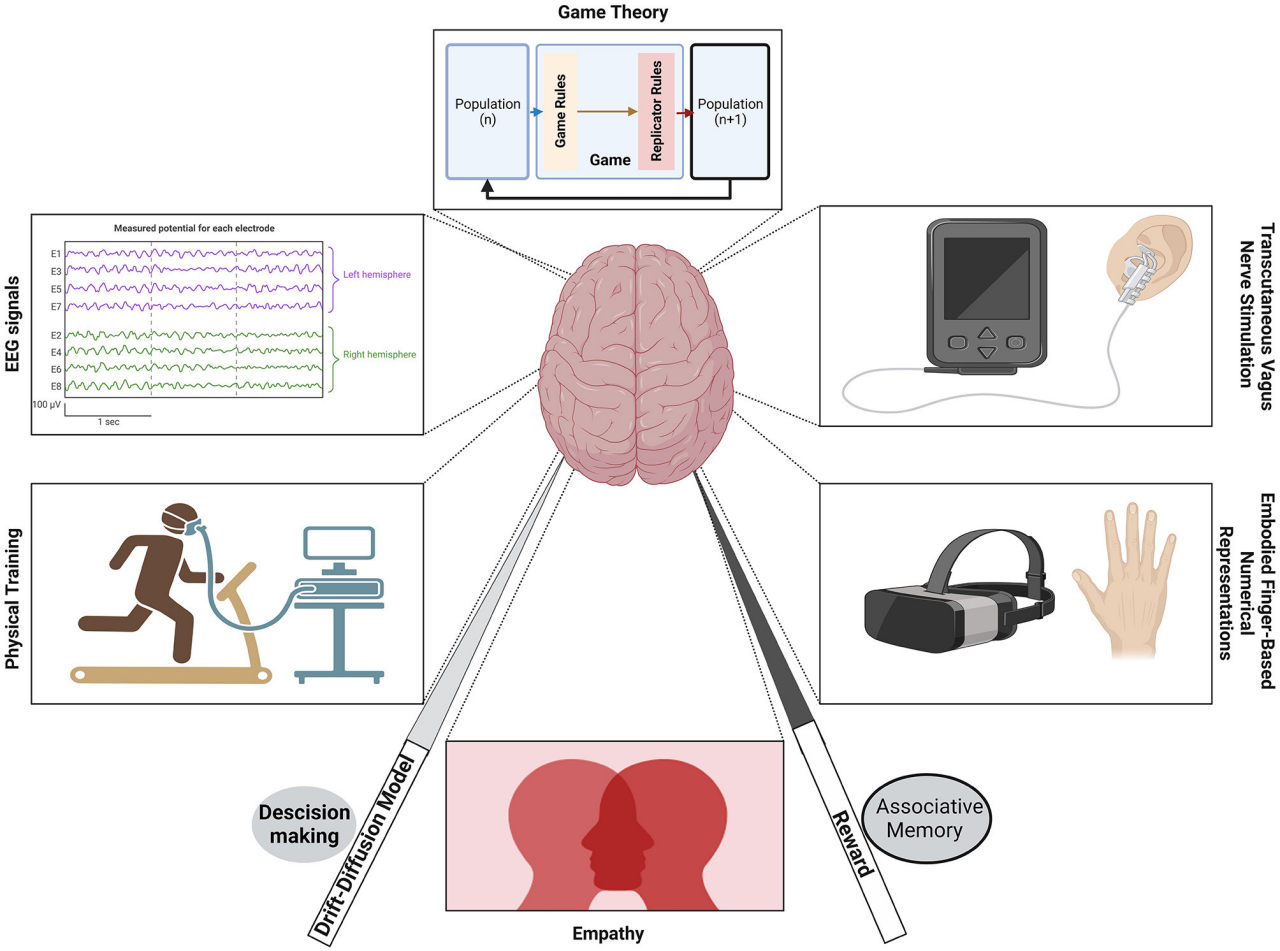
Pictorial representation of the contributions of this special volume.

Yan et al. investigated the impact of rewards on associative memory, specifically examining the influence of unitization depths. The authors utilized event-related potentials (ERPs) to examine the effects and neural underpinnings of various unitization depth and reward sets on associative memory. The main findings indicated that rewards boosted low-unitized item associative memory more.

Wang et al. explored the training and transfer effects of combining inhibitory control training with transcutaneous vagus nerve stimulation. The study showed that inhibitory control training (ICT) enhanced the performance of inhibitory control (IC). Overall, the results suggest that ICT training through tVNS can enhance IC among individuals in various professional sectors. This paper suggests novel avenues for cognitive enhancement research.

Son et al. compared the impact of motor imagery training to that of physical training on motor performance - specifically, response inhibition using the stop signal task. Fifty-one participants were divided equally into three groups - a physical training group (PT), a motor imagery training group (MIT), and a motor imagery plus physical training group (MIPT). The results showed that a training combing motor imagery and physical training was the most effective one. This research provides a framework to overcome social dilemmas in similar conditions.

Myers et al. examined the application of the drift-diffusion model (DDM) of decision-making in cognitive psychology, neuroscience, and health sciences. The paper describes the basic assumptions in using the DDM, and the way the DDM results are normally presented and evaluated. The authors illustrated how the DDM is implemented and used systematically on an example dataset so that interested researchers may find it easy to apply these techniques in their work.

Chen and Yang studied the causes of cognitive-behavioral differences between officials and folks in China's poverty alleviation program from the perspective of evolutionary game theory. The results suggested that suitable government sponsored interventions help mitigate these differences and avoid the situation becoming like a prisoners' dilemma.

de Carvalho Souza et al. examined whether finger-based representation of numbers employed in numerical and arithmetic processing in children and adults is built on simple perceptual features or accomplished through embodiment. They developed an experimental paradigm to study embodiment during a finger-based numerical task using Virtual Reality (VR) and a tactile stimulator. The results showed that their novel paradigm using VR was successful in delivering tactile stimulation to all fingers of a participant and recording the motion simultaneously. This paper presents a new methodology to study the embodiment of finger-based numerical representations and other high-level cognitive functions.

Guo et al. investigated the relationship between emotional activity and cognitive load during multimedia learning from an emotion dynamics perspective using electroencephalography (EEG) signals. They found that emotional activity was negatively associated with cognitive load. The findings have direct application in designing video lessons in changing pedagogical scenarios.

Troncoso et al. proposed an integrative theoretical and methodological framework on studying empathy based on the 5E approach (the “E” standing for embodied, embedded, enacted, emotional, and extended perspectives of empathy). The paper illustrated how a novel multimodal approach including mobile brain and body imaging (MoBi) combined with phenomenological methods and the implementation of interactive paradigms in a natural context are adequate procedures to study empathy.

We looked at the overall representativeness of global researchers who contributed to the Research Topic in this series as well as the theoretical/practical aspects of the papers. The researchers who contributed to this volume come from six major continents - Africa, Asia, Australia, Europe, North America, and South America. However, the geographical representation is not uniformly distributed as Asia accounts for 5/8 of the contributions. All the papers, except one, have multiple authors (more than two). Two papers had collaborators from three continents. This exemplifies the multi-disciplinary collaborative nature of cognitive science that has evolved from being a mere perspective to an independent discipline.

Content-wise, 50% of the papers belong to the “original research” category while the rest belong to “Methods/paradigms including case study.” One may observe that even the original papers had some novelty concerning the rationale or paradigm of the study. There is a vast diversity in theories, epistemological frameworks, and approaches among the papers, which is true to the spirit of plurality in cognitive science. Some papers used very novel paradigms and techniques while some had more practical applications. In general, the methods followed or described in these papers exhibit flexibility for possible incorporation and integration with other compatible fields of cognitive science. Most of the papers were globally appropriate and potentially testable in different cultural settings despite the limited range of participants these studies had. By and large, we believe that this special topic series has been a successful endeavor in presenting a representative glimpse of novelty and diversity in methods and applications followed in cognitive science.

## Author contributions

PP: Writing—original draft, Writing—review & editing. EP: Writing—review & editing. RK: Visualization, Writing—review & editing.

